# Integrating Cutting-Edge Technologies in Food Sensory and Consumer Science: Applications and Future Directions

**DOI:** 10.3390/foods14244169

**Published:** 2025-12-05

**Authors:** Dongju Lee, Hyemin Jeon, Yoonseo Kim, Youngseung Lee

**Affiliations:** Department of Food Science and Nutrition, Dankook University, Cheonan-si 31116, Republic of Korea; dongju042027@naver.com (D.L.); mocom4229@naver.com (H.J.); yoonseokiiim@gmail.com (Y.K.)

**Keywords:** artificial intelligence, extended reality, biometrics, IoT, sensory evaluation

## Abstract

With the introduction of emerging digital technologies, sensory and consumer science has evolved beyond traditional laboratory-based and self-response-centered sensory evaluations toward more objective assessments that reflect real-world consumption contexts. This review examines recent trends and potential applications in sensory evaluation research focusing on key enabling technologies—artificial intelligence (AI) and machine learning (ML), extended reality (XR), biometrics, and digital sensors. Furthermore, it explores strategies for establishing personalized, multimodal, and intelligent–adaptive sensory evaluation systems through the integration of these technologies, as well as the applicability of sensory evaluation software. Recent studies report that AI/ML models used for sensory or preference prediction commonly achieve RMSE values of approximately 0.04–24.698, with prediction accuracy ranging from 79 to 100% (R^2^ = 0.643–0.999). In XR environment, presence measured by the IPQ (7-point scale) is generally considered adequate when scores exceed 3. Finally, the review discusses ethical considerations arising throughout data collection, interpretation, and utilization processes and proposes future directions for the advancement of sensory and consumer science research. This systematic literature review aims to identify emerging technologies rather than provide a quantitative meta-analysis and therefore does not cover domain-specific analytical areas such as chemometrics beyond ML approaches or detailed flavor and aroma chemistry.

## 1. Introduction

Sensory evaluation is a scientific discipline that employs assessors’ five senses to identify product attributes and to measure and interpret foods both qualitatively and quantitatively [1]. This approach has evolved through the integration of psychology, physiology, chemistry, physics, and statistics, and has been systematically implemented based on standardized procedures and consistent protocols to elucidate the interactions between stimuli and human responses, as well as to predict consumer preferences and behaviors [1,2]. However, because it relies on assessors’ subjective perception, the results may vary depending on inter-individual differences, cultural background, and affective state, thereby introducing potential cognitive biases [2,3]. In addition, the recruitment, training, and management of panels for descriptive analysis require considerable time and financial resources, imposing practical constraints on the widespread implementation of sensory evaluation [3]. Consequently, in real industrial and research settings, sensory evaluation often fails to achieve adequate levels of prediction accuracy, ecological validity, and throughput.

To overcome these limitations, researchers in sensory science have explored novel approaches, and the recent incorporation of digital technologies has demonstrated considerable potential to greatly enhance the objectivity, efficiency, and scalability of sensory evaluation processes [4]. In particular, artificial intelligence (AI) and machine learning (ML), biosignal measurement technologies, extended reality (XR), and digital sensing and automation systems are being actively applied in sensory and consumer research. AI and ML enable the processing and analysis of large-scale datasets, thereby refining and enhancing the prediction of consumer preferences and behaviors, while biosignal measurement techniques—such as electromyography (EMG), electroencephalography (EEG), eye tracking, skin conductance (SC), heart-rate variability (HRV), and facial-expression analysis (FEA)—capture implicit physiological responses, thereby complementing the limitations of subjective self-report measures [5,6]. Furthermore, XR technologies including virtual reality (VR), augmented reality (AR), and mixed reality (MR) simulate realistic consumption contexts to enhance the ecological validity of sensory evaluations, whereas such digital sensing and automation technologies as the Internet of Things (IoT), robotics, electronic tongues (E-tongue), and electronic noses (E-nose) improve the precision and efficiency of data acquisition and analysis [7,8]. When used synergistically, these digital technologies can effectively address the intrinsic limitations of conventional sensory evaluation and significantly broaden its research and industrial applicability. Nevertheless, systematic reviews that synthesize sufficient evidence on the extent to which individual technologies improve specific aspects of sensory evaluation, and that clearly delineate their scope of application and limitations, remain scarce. Therefore, it is necessary to systematically examine, for each digital technology currently used in sensory evaluation, whether adequate evidence has accumulated regarding its ability to enhance key indicators—such as prediction accuracy, ecological validity, and throughput—and to clarify its practical application range and constraints.

The purpose of this review is to provide a comprehensive overview of the development and significance of emerging technologies applied in sensory evaluation. Specifically, the aim of this review is to critically synthesize the evidence on how these technologies improve prediction accuracy, ecological validity, and throughput in sensory evaluation. Specifically, this review seeks to address the following key questions.

(i) Which major digital technologies (e.g., AI, XR, biometrics, digital sensors) are currently used in sensory and consumer evaluation, and how have they been applied in sensory studies?
(ii) How do these technologies differ in their structural and functional characteristics, and in what ways do they improve key indicators in research and practice, such as predictive power, validity, and efficiency?
(iii) How can the individual and integrated use of these technologies, together with sensory software, enhance resource-management efficiency and evaluation objectivity, and what ethical and practical issues arise in this process?

**Figure 1:**
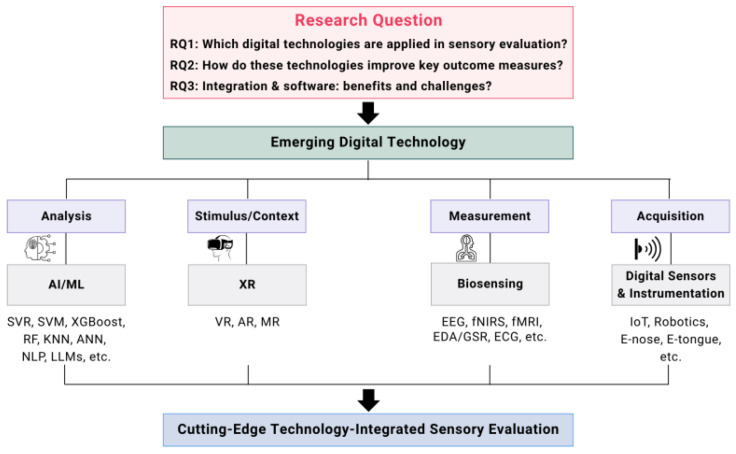
Overall workflow for cutting-edge technology-integrated sensory evaluation.

In this review, we synthesize recent research trends on AI, XR, biosignal measurement, and digital sensing technologies, and critically discuss the new possibilities these technologies offer for data analysis and interpretation. In addition to highlighting the roles of individual technologies, this review emphasizes the importance of their integrated application, examines the potential of sensory software, and explores strategies to enhance both efficiency in resource management and objectivity in evaluation. Moreover, it addresses ethical considerations associated with the adoption of these technologies and proposes future directions for the advancement of sensory science. Figure 1 schematically illustrates the overall conceptual framework of this review.

## 2. Methodology

In this study, we conducted a systematic literature review to synthesize and critically evaluate previous work applying cutting-edge digital technologies to sensory evaluation, consumer research, and related food studies. We searched major databases (Scopus, PubMed, Google Scholar) for articles published between 2015 and 2025 using combinations of sensory- and consumer-related terms (e.g., “sensory evaluation”, “consumer test”, “food”) and technology-related keywords (e.g., “artificial intelligence”, “machine learning”, “extended reality”, “biometrics”, “digital sensor”, “internet of things”). Targeted searches based on key terms and seminal papers identified in the initial search were additionally performed, and the reference lists of preliminarily eligible articles were screened to capture further relevant studies.

After removing duplicate records, studies were included if they (i) involved food or beverage products or food-related sensory stimuli, (ii) reported primary outcomes on sensory responses, liking, choice behavior, or food-quality monitoring and management, and (iii) incorporated at least one of the following technologies into the study design: AI, ML, XR, biometric measurements, digital sensors, or the IoT. Studies were excluded when the methodology was insufficiently described, the article was not written in English, or full-text access was not available, preventing adequate evaluation.

For the studies that met the inclusion criteria, we used standardized extraction forms to collect information on the study context, food category, technology applied, main objectives and outcomes, and implications for sensory and consumer science, and organized these data into tables by technology type. Based on this dataset, we compared research aims and key findings across technology categories, examined the potential for integrated use of these tools in sensory, consumer, and food-quality research, and evaluated related software tools as well as the methodological and ethical issues, advantages, limitations, and application domains of each technology, thereby identifying major gaps that warrant further investigation.

## 3. Advances in Technologies for Sensory and Consumer Sciences

### 3.1. Artificial Intelligence and Machine Learning

Artificial intelligence collects vast amounts of data and learns complex patterns through processes of rule learning and generalization [9,10]. In consumer sensory science, AI functions as a powerful analytical tool that can predict food taste and elucidate the interaction mechanisms between consumer preferences and the sensory attributes of foods [9,10]. As the food industry continues to advance and large volumes of product-related data are accumulated throughout food quality development processes, the necessity and significance of applying AI for food quality prediction have become increasingly recognized [11,12].

In recent years, research employing AI–based ML techniques has grown rapidly. ML has been utilized not only for predicting food quality but also for elucidating the correlations between consumer preferences and product sensory characteristics, as well as for developing predictive models that can complement or even substitute for human sensory evaluations. Moreover, with the increasing prevalence of natural language processing (NLP)–based large language models (LLMs), several studies have demonstrated their potential applications in product development through the analysis of consumer-generated text reviews.

**Figure 2:**
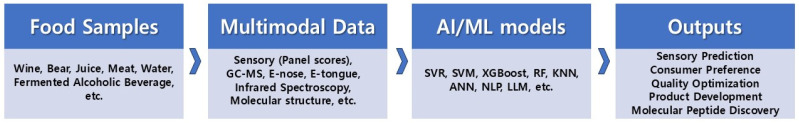
AI and ML in food sensory evaluation.

More recently, the integration of molecular docking techniques with ML has been actively explored for the identification of peptide candidates capable of mimicking or replacing specific taste sensations. Therefore, these advances suggest that AI is becoming an indispensable tool in consumer sensory science. Figure 2 presents the application mechanisms of AI and ML models in this field.

#### 3.1.1. Machine Learning Approaches for Sensory and Quality Prediction

Machine learning, a subfield of AI, learns highly complex patterns within large datasets and identifies relationships among data through training and testing processes. Owing to its high predictive accuracy, ML has become a powerful alternative to traditional predictive models, such as partial least squares (PLS) regression and Bayesian statistics, in the field of consumer sensory science [13,14]. Recently, a variety of ML algorithms—including Support Vector Regression (SVR), Support Vector Machine (SVM), Extreme Gradient Boosting (XGBoost), Random Forest (RF), K-Nearest Neighbors (KNN), Artificial Neural Networks (ANNs), and Deep Learning (DL)—have been applied to predict and classify sensory attributes and food taste [11,15,16]. Among these, SVM and SVR utilize kernel functions to process both linear and nonlinear data. SVM has shown excellent performance in classification problems involving small-sample and high-dimensional pattern recognition, while its extended variant, SVR, also demonstrates strong capability in regression analysis [17,18].

Random Forest, a bagging-based learning model, predicts complex patterns by training multiple decision trees and effectively prevents overfitting, thereby exhibiting excellent performance in classification tasks. In contrast, XGBoost, an ensemble algorithm that combines tree-based learning with gradient boosting, is widely applied to both classification and regression problems and is recognized for its relatively high predictive accuracy [14,18,19]. KNN, an instance-based learning algorithm, effectively captures data similarity without the need for complex training processes and can be utilized for both classification and regression. Meanwhile, ANN, with its multilayered neural architecture, is capable of learning nonlinear and complex data patterns, making it suitable for pattern recognition, classification, and regression tasks [18]. DL, a subfield of ML, is an AI technique that automatically learns key features from large-scale datasets and achieves high accuracy in data classification and prediction [11,15].

Table 1 summarizes the key findings of previous ML-based studies across various food categories and the ML algorithms employed. It also presents the data size used for model development, the risk of overfitting, and the validation protocols adopted in each study.

As shown in Table 1, ML technologies have consistently demonstrated high predictive accuracy across various food categories, reinforcing their growing applicability in modern sensory evaluation research. By providing objective and rapid insights from large-scale datasets, ML serves as a promising complement to traditional sensory assessment. Recent studies particularly highlight the effectiveness of hybrid ML models and diverse validation protocols, which help reduce overfitting and enhance model stability as well as classification and prediction performance [14]. Consequently, these findings indicate that ML is rapidly expanding its role across the food industry and is becoming a key technological component in sensory and consumer research.

#### 3.1.2. Text Mining-Based Natural Language Processing and Large Language Models

In food choice behavior, taste serves as a primary motivational factor for consumers; however, identifying the taste of foods and the degree of liking from consumers’ language is limited using unstructured and ambiguous expressions, making it difficult to achieve a systematic understanding of taste through conventional consumer surveys [24]. Therefore, for food companies to discover new trends and gain a competitive advantage, it has become essential to collect vast amounts of consumer-related data through web-based platforms [25]. As one of the efficient and rapid AI-based approaches for this purpose, text mining and NLP have emerged as key trends in sensory science in recent years [25]. NLP, a subfield of text mining, grounded in AI, computer science, and linguistics [24,26]. In addition, it represents a branch of AI that systematically analyzes language through text preprocessing, syntactic structures, and semantic analysis, enabling the interpretation of consumers’ free-form or ambiguous textual expressions [24,26]. LLM are DL–based models trained on massive amounts of natural language text and images to generate human-like text outputs, with representative examples including GPT-4, ChatGPT-5.1, BERT, Claude, and Gemini [27,28,29]. Table 2 summarizes the key findings of previous studies that employed NLP-based LLMs, as well as the AI and ML algorithms utilized in those studies.

Recently, in the field of sensory evaluation, data mining for recipe development and consumer feedback analysis have been applied to food flavor characterization and product development [24,26,30]. In particular, analyses focusing on consumers’ emotions not only provide insights into product perception but also enable the prediction of consumer preferences, demonstrating increasing applicability and expansion in sensory and consumer research [24,26,30]. In the previous studies summarized in Table 2, the adoption of text-mining-based NLP and LLM approaches demonstrated potential for consumer-driven data analysis; however, their predictive accuracy remained somewhat lower compared to traditional ML models. Therefore, future studies should focus on developing methodological approaches that enhance the reliability and robustness of these models to facilitate their practical implementation and commercialization.

#### 3.1.3. Molecular Dynamics Simulation

Molecular Dynamics simulations play a crucial role in explaining the fundamental molecular mechanisms and the interactions between peptides and enzymes, while molecular docking serves as a molecular simulation technique that elucidates interaction mechanisms by calculating the binding sites between peptides and receptors based on receptor structures [16,33]. Traditional methods for screening peptides that contribute to specific taste sensations—such as sensory evaluation combined with filtration or chromatography techniques like High Performance Liquid Chromatography—are often time-consuming and costly [34,35]. Therefore, recent studies in the field of sensory evaluation have actively employed approaches that integrate peptide extraction from food sources with ML-based peptide screening and molecular docking to identify peptides contributing to specific taste perceptions. Table 3 summarizes the input data and ML models utilized in previous studies related to Molecular Dynamics (MD) simulations.

The previous studies summarized in Table 3 demonstrate that integrating ML with MD simulations enables rapid processing of complex peptide-screening procedures that traditionally required substantial time and effort, thereby accelerating the commercialization of bioactive or taste-contributing peptides. Moreover, ML-based MD simulation research in sensory evaluation has been increasingly active in recent years, and the scope of molecular-level investigations in sensory science is expected to further expand in the future.

#### 3.1.4. Limitations of Artificial Intelligence and Machine Learning for Sensory and Consumer Science

Artificial intelligence technologies are emerging as an efficient alternative to traditional sensory evaluation methods. In particular, the use of ML has been progressively expanding across the food industry in recent years, including applications in MD simulations and molecular docking studies. However, the utilization of AI in this field still faces several challenges and limitations.

In ML models, as dataset size increases, issues such as overfitting may arise, which can diminish model accuracy [39]. To address these limitations, challenges related to data cleaning, missing values, class imbalance, data leakage, and noise must be resolved [40,41,42,43]. This requires appropriate data preprocessing procedures, including rebalancing, data transformation, normalization, and outlier removal [40,41,42,43]. Furthermore, a systematic validation protocol is essential for evaluating model performance and reliability. In the study by [44], external validation was performed to assess ML performance. The results showed that the model’s predictions aligned with actual human sensory evaluation outcomes within an acceptable error range, underscoring the importance of external validation in ensuring the reliability and stability of ML models [44].

Additionally, real-world data—including consumer liking data—can experience domain shift and drift over time due to changes in data patterns and environmental conditions [39]. These shifts can degrade a model’s predictive performance, making regular performance monitoring and periodic recalibration essential for maintaining generalization capability, stability, and high predictive accuracy [39].

Finally, when data collection relies solely on a single method, the classification and prediction accuracy of ML models may decrease. To overcome these limitations, the integration of diverse data sources—including electronic sensing devices (E-nose, E-tongue), spectroscopic methods, human sensory evaluation, and physicochemical indices—is essential. Moreover, combining multiple predictive models rather than relying on a single ML model can further enhance the reliability and robustness of the analytical outcomes.

In recent studies on text mining–based sensory evaluation technologies, ref. [31] reported that ChatGPT provided positive evaluations even for hypothetical products formulated with ingredients that may evoke somewhat negative consumer reactions. This finding suggests that LLMs may interpret product attributes differently from actual consumer perceptions, highlighting the necessity of human-based validation to ensure the reliability of such interpretations. Furthermore, ref. [31] emphasized the importance of consistency in the prompt environment and the standardization of evaluation procedures. This indicates that prompts should be structured in a uniform and consistent manner, and that clear instructions are essential for obtaining stable results [31].

According to [26], online review authors do not fully represent the broader consumer population and are often limited to specific linguistic and cultural groups. This implies that linguistic and cultural differences may introduce bias. Because web-based platforms include consumers from diverse backgrounds, linguistic and cultural variability, as well as the representativeness of consumer samples, must be carefully examined [9,26,28].

Although AI-based digital technologies continue to advance, clear limitations remain in fully understanding and analyzing consumer data that are rooted in complex and multidimensional emotions and experiences. To reduce the risk of model bias stemming from skewed training data, AI systems must incorporate a wide range of societal perspectives and information, and sufficient human verification is required to prevent the generation of misleading outputs [31,45]. Therefore, an integrated approach that combines AI-based analysis with human sensory data is essential.

### 3.2. Extended Reality: Virtual, Augmented and Mixed Reality

Food choice is the outcome of a complex interplay of factors and has long been treated as a major research topic in the fields of sensory and consumer science [46]. Recently, particular attention has been directed to the influence of consumption context on consumer behavior and on the perception of foods and beverages, and contextual cues—such as place of consumption, social situation, and auditory stimulation—have been reported to induce significant changes in perception and choice [46]. In line with the growing emphasis on these contextual factors, the fields of sensory and consumer science have actively pursued studies that apply immersive technologies—VR, AR, and MR—to reproduce real consumption situations [47]. This approach enables consumers to experience foods and beverages across diverse environments and contexts, thereby complementing the limitations of conventional sensory evaluation and providing an assessment environment that secures both internal validity and external validity [48].

Moreover, XR-based evaluation environments may represent a realistic alternative from a cost perspective. According to [49], studies conducted in real consumption settings required approximately 150% of the cost of tests performed in an already established sensory laboratory, whereas the relative cost of immersive consumption environments decreased as sample size increased, reaching about 125% of the laboratory cost at *n* = 120. These findings suggest that, despite the initial investment required for system setup, immersive environments can be more cost-efficient than traditional real-life consumption settings and, in consumer studies with sufficiently large sample sizes, may serve as an alternative or complementary tool to conventional sensory laboratories.

**Figure 3:**
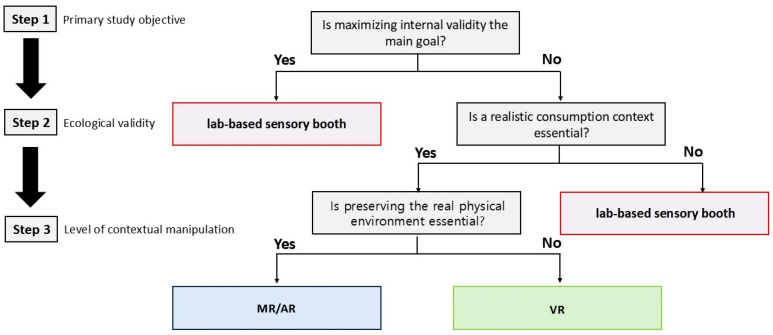
Conceptual decision tree for selecting lab and XR-based (VR, MR/AR) test environments in Sensory evaluation.

Against this background, the present review proposes a conceptual decision tree (Figure 3) to guide the selection of an appropriate test environment—conventional laboratory, VR, or AR/MR—for sensory and consumer evaluation. When the primary objective is to ensure high internal validity, a laboratory-based sensory test is most appropriate, whereas XR environments should be considered when the goal is to enhance external validity by incorporating realistic consumption contexts. Within XR, AR/MR-type hybrid settings are preferable when maintaining the real physical space, while overlaying limited virtual elements is desired, whereas fully virtual, VR-based environments are better suited when fine-grained control of contextual cues such as lighting, spatial layout, and social interactions is required.

**Figure 4:**
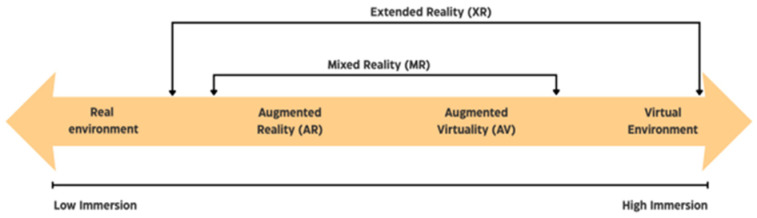
Spectrum of reality–virtuality: representation of XR, VR, AR, and MR (Adapted from [51], with permission from IEICE; Copyright ©1994 IEICE).

Extended reality, also referred to as cross reality, is a concept that encompasses immersive technologies such as VR, AR, and MR, as well as human–machine interaction based on these technologies, metaverse environments, and spatial computing [50]. XR spans the continuum between real and virtual environments and realizes the convergence of the physical and digital worlds (Figure 4).

Virtual reality is a technology that, through a head-mounted display (HMD) that occludes the user’s field of view, replaces the real environment with three-dimensional or virtual imagery to provide a fully immersive experience [52]. In sensory evaluation, VR enables consumers to experience foods and beverages within a virtual environment, thereby allowing observation of perception and responses in contexts that approximate real-world conditions, while simultaneously imparting a sense of presence that supports interaction with the virtual environment [52]. AR is a technology that superimposes digital imagery or information onto physical space by means of devices such as smartphones, tablets, AR glasses, and headsets [53]. Through this approach, users can perceive virtual elements while remaining aware of their surrounding environment, and not only visual images but also auditory stimuli—such as spatial audio—can be combined with the real environment [54].

In augmented reality, interaction between the user and virtual stimuli is possible; however, unlike VR, direct interaction between virtual stimuli and elements of the real environment is limited [55]. MR is a concept situated between AR and VR, providing an environment in which real and virtual elements coexist and can interact in real time [56]. Users, wearing an HMD or AR glasses, can perceive physical elements—such as their hands and arms or evaluation samples—as integrated within the virtual scene and can manipulate virtual stimuli as if they were part of the physical environment, thereby affording a high degree of realism [53,55]. Table 4 summarizes the overall differences among VR, MR, and AR.

Extended reality presents new avenues for application in sensory evaluation and affords research opportunities to investigate consumer perception and behavior with greater precision. Notably, among XR-based immersive technologies, VR has been most actively applied, and recent prior studies have been reported in this domain. Ref. [58] demonstrated that enjoyment and purchase intention were significantly higher when virtual foods matched their real counterparts; this cross-reality effect was particularly salient for foods with delicate taste attributes and low familiarity, thereby evidencing that digital aesthetic cues can influence consumer perception and behavior. Ref. [59] reported that an immersive and realistic VR context elicited positive affective responses and increased beer acceptability, and machine-learning analyses identified affective responses as an important predictor of acceptability.

In a recent AR-based study, ref. [54] confirmed, using lasagna and strawberry yogurt, that visual- and auditory-based AR tools provide consumers with novel and memorable experiences; however, they further reported that nutritional information exerted a greater influence on changes in product perception irrespective of the presentation format. Ref. [60] showed that AR facilitates consumers’ mental simulation, thereby increasing desire for food and purchase likelihood, with these effects mediated by enhanced perceptions of personal relevance.

In a mixed reality study, ref. [56] found that congruency between product and contextual color/shape strengthened sourness perception; participants tended to associate red/pink with sweetness and green/yellow with sourness, and to link rounded shapes with sweetness and angular shapes with sourness, confirming MR as a useful tool for controlling visual cues to investigate taste perception. Ref. [61] demonstrated that sensory evaluation can be conducted in MR while maintaining immersion by employing a gesture-recognition-based virtual questionnaire and a customized wine glass.

Moreover, the implementation of a realistic virtual bar via audio and avatars, together with real-time gesture responses and wine-glass interactions, enabled multiple participants to provide data concurrently under full immersion. Collectively, these studies indicate that VR, AR, and MR technologies extend beyond mere contextual replication in sensory evaluation and open possibilities for the integrative investigation of consumer perception, cognition, and behavior—including purchasing behavior, perceptions of nutritional information, choice behavior, and crossmodal effects. In addition, Table 5 summarizes prior studies applying VR, AR, and MR technologies in sensory evaluation and presents the principal findings of each study.

Immersion, a core element of XR technologies, is a critical factor that separates users from the real environment and enables them to experience vivid and wide-ranging virtual worlds [71]. An effective immersive experience engenders a sense of presence—as if users exist in a world analogous to reality—which plays an important role in narrowing the gap between laboratory contexts and actual consumption contexts [74]. Presence is the user’s subjective perception of immersion, and it strengthens as the level of immersion increases [74]. High levels of immersion in VR, particularly when accompanied by mismatches between visual and bodily sensory cues, can induce cybersickness—manifesting as nausea, dizziness, headache, and eye strain—which, in the context of sensory evaluation, may reduce participants’ concentration, distort their responses, and increase the risk of dropout [75]. Accordingly, recent XR-based sensory studies have employed standardized questionnaires such as the igroup presence questionnaire (IPQ), system usability scale, and independent television commission-sense of presence inventory to assess immersion and presence and have used the Simulator Sickness Questionnaire (SSQ) to quantify cybersickness. Several studies have reported significantly higher presence in 360° VR and context-evoking conditions than in conventional laboratory or traditional control settings [7,66,76]; in particular, because the IPQ is rated on a 7-point scale, factor scores at or above the midpoint of 3 can be interpreted as reflecting a reasonably sufficient level of presence. Moreover, XR-based sensory-evaluation environments should be designed and managed such that total SSQ scores preferably remain below 10, corresponding to negligible or minimal levels of cybersickness [65].

In summary, XR-based sensory evaluation is a novel approach that helps overcome limitations in internal and external validity between traditional sensory booths and real consumption environments. By employing VR, AR, and MR in line with specific research objectives, it enables the design of consumption contexts that are difficult to realize with conventional methods, and its applications are increasingly extending across the food domain.

There are several limitations inherent in applying XR technologies to sensory evaluation. First, wearing equipment such as HMDs can induce fatigue during prolonged experiments and, for some participants, physiological discomfort such as cybersickness [77], highly immersive media can markedly increase participants’ information-processing demands, potentially leading to cognitive overload, which may distort intrinsic sensory responses or reduce attentional focus [75]. Third, at early stages of exposure—particularly among users unfamiliar with virtual environments—a novelty effect may arise during adaptation to new interaction modalities, imposing additional cognitive burden and thereby lowering experimental reliability [78].

Finally, the costs of equipment and system deployment are high, and practicality for application in large-scale consumer studies remains limited. Therefore, to address these constraints and expand the applicability of XR in sensory evaluation, further research is needed to pursue improvements in equipment, the refinement of measurement indices for immersion and presence, the implementation of multisensory stimuli, and strategies for scaling to large-sample consumer research.

### 3.3. Biometrics and Physiological Measurements

Traditional sensory evaluation has relied on respondents’ subjective reports, which has limitations as it is influenced by various factors [79]. Therefore, research combining existing sensory evaluation protocols with emerging technologies has been conducted to more comprehensively reflect consumer responses and provide deeper insights [2], and the number of such studies is steadily increasing [80]. Among these innovations, biometrics has emerged as a particularly promising approach [1]. Biometric data serve to provide additional information beyond explicit measurement data [81]. Biometrics is defined as “automated recognition of individuals based on their biological or behavioral characteristics” [82].

Biometric technologies utilized in sensory evaluation include EEG, functional near-infrared spectroscopy (fNIRS), functional magnetic resonance imaging (fMRI), electrodermal activity/galvanic skin response (EDA/GSR), electrocardiography (ECG), respiration, blood pressure, FEA, and EMG [80,83,84]. This section classifies the major biometric technologies applied in sensory evaluation by type and summarizes recent research trends.

#### 3.3.1. Nerve and Brain Activity

Electroencephalogram signals are collected by measuring the potential difference between the active and reference electrodes [85]. Although the specific frequency ranges vary across studies, the recorded EEG signals are generally classified into five frequency bands: delta, theta, alpha, beta, and gamma [86]. Unlike early approaches that required drilling or perforating the skull to place electrodes, modern techniques allow the acquisition of high-quality EEG data through non-invasive methods. Furthermore, research has been conducted to develop smaller and wearable wireless EEG devices for practical applications [87]. According to [88], dry EEG systems provide a solution to overcome the limitations of wet EEG systems, which are unsuitable for long-term measurements, and the use of dry and wireless EEG systems is expected to increase. Compared to other neuroimaging techniques, EEG is considered a promising neuroscientific tool due to its relatively low cost and easy accessibility of devices for research purposes [89].

Functional near-infrared spectroscopy is defined by Marco [90] as a “neuroimaging technique for mapping the functional activity of the human cerebral cortex.” It is a non-invasive and safe technology that uses laser diode or light-emitting diode light sources to deliver near-infrared light between the source and the detector through biological tissues. In addition, fNIRS has the advantage of being able to utilize relatively inexpensive, portable, and wireless devices compared to fMRI. fMRI is a technique that detects changes in the blood oxygenation level-dependent (BOLD) signal of MRI that arise from alterations in brain states induced by stimuli or tasks. Furthermore, fMRI is known as a useful tool capable of extracting new information from brain systems associated with complex responses through simple activation maps obtained under various experimental conditions [91].

Ref. [92] compared consumer survey responses with EEG results on citrus flavor and identified correlations, confirming consumers’ unconscious reactions to citrus flavor that were not revealed through self-reported evaluations. The study by [93] is noteworthy for providing the first insights into basic taste sensitivity using fNIRS and for exploring the predictability of consumer preference. In this study, consumer preference data for two types of chocolate were obtained through subjective evaluations using a 7-point Likert scale, while participants’ unconscious responses were measured by recording oxyhemoglobin (OxyHb) concentrations through an fNIRS headband. Regarding basic taste, neural activity tended to decrease in response to sweetness and increase in response to bitterness. In terms of preference, participants were classified as chocolate likers and dislikers, and a significant difference between the two groups was observed at channel 16. This study demonstrated that responses in certain prefrontal channels could predict consumer preferences, suggesting that fNIRS holds potential as a complementary tool for future consumer evaluation research.

Building on these studies, the measurement of neural activation related to sweet taste perception using fNIRS was further investigated in depth by Jiayu [94]. In this study, the perceived sweetness intensity and emotional valence of three concentrations of sucrose solution were evaluated using a 15-point scale and a 9-point scale, respectively. As the sucrose concentration increased, the number of significantly activated channels rose from seven to eleven, indicating an expansion of brain activation areas. The classification accuracy of positive emotional responses identified by fNIRS was approximately 72%.

In contrast, the composite similarity index of the implicit–explicit regression model showed high overall consistency, with values of 0.998 for sweetness intensity and 0.889 for emotional valence, suggesting that fNIRS could serve as a supplementary tool in consumer evaluation. The study further proposed that future research should address temporal–spatial resolution by expanding channels or integrating fNIRS with EEG and fMRI and verify the classification potential for other basic tastes. Additionally, the latest studies employing EEG, fNIRS, and fMRI technologies in consumer sensory evaluation are summarized in Table 6.

#### 3.3.2. Autonomic Nervous System Responses

**Figure 5:**
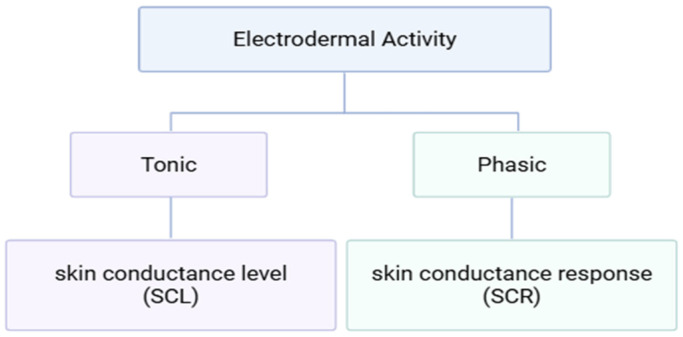
Classification of EDA into SCL and SCR.

Indicators of autonomic nervous system (ANS) responses include EDA, HR, HRV, and skin temperature (ST) [107]. GSR is mostly used interchangeably with EDA [108] and is one of the most widely utilized and well-established recording methods and biosignals [109]. The EDA commonly used in research is typically measured using direct current (DC) or constant voltage methods with silver/silver chloride (Ag/AgCl) electrodes and sodium chloride or potassium chloride electrolytes [110]. In exosomatic recording methods, when the voltage is held constant, the signal is recorded in units of skin conductance (SC), whereas when the current is held constant, it is recorded in units of skin resistance (SR) [110]. The level of sympathetic nervous system activity can be assessed through variations in skin conductance, which reflect physiological responses of the organism corresponding to cognitive and emotional states of the central nervous system [108]. Depending on the temporal dynamics of change, skin conductance is categorized into tonic activity, referred to as skin conductance level (SCL), and phasic responses, referred to as skin conductance response (SCR) (Figure 5) [111].

Electrocardiography is a technology that records cardiac signals with the highest degree of accuracy, and the voltage deflections that constitute the ECG correspond to the electromechanical phenomena of the heart and are represented by five letters [112]. Among ECG indices, heart rate (HR) generally indicates the number of heartbeats within 60 s [112], while heart rate variability (HRV) represents an immediate response of the autonomic nervous system to detected stimuli [113]. In particular, GSR and HRV sensors are among the most widely distributed biofeedback devices [114].

Alessandro [115] measured ECG and GSR signals associated with red wines of varying “emotional power” to identify differences in autonomic nervous system activity. The study found that as positive judgments toward wine increased, GSR characteristics (total GSR, tonic GSR) also increased, and significant emotional differences were observed in ECG parameters such as HR, Standard Deviation of Normal-to-Normal intervals (SDNN), cardiac sympathetic index, and NN50. These findings support the hypothesis that specific foods or beverages may serve as drivers of positive dietary behavior and suggest the potential for expanded studies with more diverse sample groups.

Ref. [116] examined the effects of HR, HRV, skin conductance, and frontal alpha asymmetry in response to solutions differing in both individual and general levels of preference. As a result, significant differences were observed between preferred and non-preferred beverages in HR and skin conductance, particularly in latency, while no significant differences were found in frontal alpha asymmetry. The study by [117] measured the SCR for samples with sweet, bitter, and astringent tastes and analyzed SC using implicit testing methods, exploring its potential as an auxiliary tool for understanding individual differences in sensory responses. Furthermore, the results suggested that SCR measurement can provide valuable insights into the relationship between physiological responses and gustatory perception.

Ref. [118] Facial emotion recognition (FER), GSR, and cardiac pulse measurements to predict consumer acceptability for chewing gums representing five basic tastes. The study introduced a system capable of predicting consumer acceptability of new food products and proposed a novel artificial intelligence–based approach combining facial emotion recognition and biosignal data. It was confirmed that FER alone was limited in predicting consumer acceptability, whereas prediction accuracy improved when GSR and cardiac pulse were incorporated. Among these measures, GSR was identified as the most significant variable for predicting consumer acceptance. Additionally, the latest studies utilizing EDA/GSR, ECG, and ST measurement technologies in consumer sensory evaluation are summarized in Table 7.

#### 3.3.3. Eye Movements and Visual Responses

When consumers purchase food products in stores—whether in physical markets or online shops—the visual features of the products serve as crucial determinants [125]. Consequently, efforts have continued to investigate consumers’ visual responses to various stimuli [126]. Eye-trackers used for gaze tracking are defined as “devices used to estimate the direction of the eyes relative to the position of the head or gaze.” There are several types, including video-based eye trackers, electro-oculography, and scleral search coils [127], and they are generally classified into two main types: desktop-based and mobile-based systems [126].

Eye-tracking measures several parameters such as pupil dilation, smooth pursuit, microsaccades, saccades, blinks, and fixations [128]. Most studies in sensory and consumer science utilizing eye-tracking have focused primarily on fixation characteristics, which are interpreted as indicators of visual attention [126]. Eye-tracking technology determines the position of the gaze on a screen based on the pupil center and corneal reflection produced by infrared light and is regarded as the most accurate non-invasive method for measuring gaze position [81].

Ref. [129] employed the eye-tracking method to measure participants’ gaze patterns toward lemon-based food images under repeated olfactory stimulation with lemon scent. The results showed that the scent continuously directed participants’ visual attention to the product; however, under repeated exposure, while preference for the scent remained stable, its influence on product choice diminished. Ref. [130] found through eye-tracking that nutritional information attracted the highest visual attention, yet the main factor influencing purchase intention was the “Product of New Zealand” logo. This finding indicated that the orange juice package attracting the most visual attention was not necessarily the most preferred product.

In the study by [131], eye-tracking and preference evaluations were conducted simultaneously using high-calorie and low-calorie food images. Participants recognized high-calorie foods more quickly and maintained longer visual attention on them. The research by Savannah [132] demonstrated that consumers’ pupil responses statistically mediated the relationship between assortment size and product preference in display shelves, revealing that larger assortments led to pupil constriction and reduced cognitive processing capacity. Moreover, pupil size varied in the same direction as the price level of the chosen option but in the opposite direction of familiarity. Additionally, the latest studies utilizing eye-tracking technology in consumer sensory evaluation are summarized in Table 8.

Ref. [137] emphasized the importance of using advanced technologies to measure emotional responses in order to better understand consumer behavior and decision-making. Facial emotion recognition technology, which enables the identification of emotional expressions, can measure both cognitive and affective characteristics, particularly the degree of positive or negative emotions perceived by the participant [138]. Moreover, facial expression (FE)s serve as indicators of immediate emotional responses elicited by sensory stimuli such as the taste, texture, and appearance of food. The process of food consumption can be observed and recorded in real time, allowing specific emotions to be quantitatively analyzed [139]. According to [140], EMG technology analyzes the activity of specific facial muscles or muscle groups and classifies the measured facial behaviors into distinct emotions based on activation patterns. EMG records muscle activity by attaching electrodes to the skin surface, enabling observation of muscle activation associated with particular emotions [141].

Ref. [142] analyzed FE within the context of consumer product choice and measured the emotions elicited using FEA. The study demonstrated that using FE data improved the prediction of consumers’ product choices compared to relying solely on a single hedonic scale. Furthermore, by examining the role of emotions in the selection of credence goods such as organic and vintage wines, the study clarified the relationship between consumer emotions and product choice. For future research, it was suggested to identify the optimal method for emotion measurement and to explain why positive emotions influenced the selection of only specific wine attributes, which was further extended by [143]. In their study, consumer emotional responses to white, red, and port wines were distinguished using FaceReader, confirming that the type of wine significantly influenced the formation of emotional responses.

The study by [144] is noteworthy in that, unlike previous research which mainly analyzed single time points or averaged data, it investigated the dynamic correspondence between subjective evaluations and facial responses using EMG signals during meals. The research reflected hedonic experiences with temporal profiles occurring during the consumption of three types of gel-based foods. A negative correlation was observed between dynamic value ratings and electromyographic signals of mastication-related muscles, demonstrating that facial EMG signals can predict hedonic responses during eating.

In the study conducted by [145], EMG was measured while participants consumed five types of jellies representing sweet, salty, sour, bitter, and umami tastes, to examine muscle response tendencies according to taste. The results showed that the taste of the jelly influenced muscle synergy and symmetry, with marked differences observed for certain taste stimuli. Although the sample size was small (*n* = 5) and statistical significance was not achieved, the findings suggested an interaction between taste perception and muscle activity, thereby providing a foundation for future sensory perception research utilizing muscular responses. Additionally, the latest studies employing FEA and EMG technologies in consumer sensory evaluation are summarized in Table 9.

#### 3.3.4. Limitation of Biometric and Physiological Measures in Sensory Evaluation

Various limitations arise when applying biometric technologies in sensory evaluation. Participants may unconsciously recognize that they are being continuously monitored by researchers, which can influence their behavioral and emotional responses [155,156]. Studies have shown that both mental and physical stress factors can affect biometric indicators. Moreover, in many studies, participants experience movement restrictions during biometric measurement due to chinrests [157,158] and electrode channels [159]. In some experiments involving biometric measurements, participants are instructed to minimize head and hand movements, keep their head fixed, and maintain a static or seated posture [102,109,141,160].

Such strict limitations on body movement can hinder participants from consuming food and beverages in a natural and unrestricted manner [93]. Additionally, the size and fixed nature of wired measurement equipment can restrict its usability and experimental environment [109,161]. Consequently, concerns have been raised about the ecological validity of such studies, as the testing environment often differs from actual consumer consumption contexts.

Another limitation is that physical movements during food consumption can generate artifacts, leading to increased data loss. One study found that jaw clenching and biting behaviors produced the most severe artifacts in scalp and ear electrodes [162]. Furthermore, consumer behavior studies utilizing biometric technologies are often limited by small sample sizes [163]. Ref. [156] reported that the number of participants who could be measured simultaneously was restricted due to equipment limitations. Another issue is susceptibility to external factors. According to [79] in food-related studies using biometric measurements, variables such as the type of product evaluated and the cultural background of participants or panels may influence the results.

### 3.4. Digital Sensing Technologies: IoT, Robotics, and Electronic Sensing Systems

#### 3.4.1. Application of Robotics Technology in the Food Industry

With the advancement of robotics technology, research on the integration of robotics into the food industry has been actively increasing [164]. In the food industry, multiple processes are involved from the initial product concept development stage to the final product release, and the incorporation of robotic technologies can be applied at each stage. In particular, at the sensory evaluation stage—prior to product manufacturing—the combination of human sensory testing with robotic technology can enhance the objectivity and accuracy of sensory data. Furthermore, the application of robotics in the final production stage offers several advantages, including ensuring consistent product quality, reducing production time, and improving operational efficiency. Table 10 presents previous studies related to robotics technologies applied in the food industry and modern sensory evaluation techniques.

According to the previous studies summarized in Table 10, the use of robotic technologies has emerged as an important complementary tool that supports and enhances human roles in sensory evaluation research and the food industry. Recently, the use of robots to replace human labor has been increasing in food-related service sectors such as cafés, cafeterias, and restaurants. Consequently, human–robot interaction has been expanding its utility across various aspects of modern sensory evaluation, product development, and overall food industry operations [164].

#### 3.4.2. Integration of Electronic Sensory Sensors and the Internet of Things

Quality control and improvement in the food industry are essential factors for the production of high-quality foods, and in recent years, the IoT framework—connecting physical objects through the internet—has been actively utilized for this purpose [167]. IoT enables the collection of sensory data through electronic sensing devices such as E-nose and E-tongue as well as physical and chemical sensors, allowing real-time data analysis and feedback to automate the system [168]. This integration simplifies many complex procedures required during food production, offering advantages such as reducing time and cost while enhancing operational efficiency [168]. Table 11 presents previous studies that have applied IoT technologies in the food industry and consumer sensory science.

**Figure 6:**
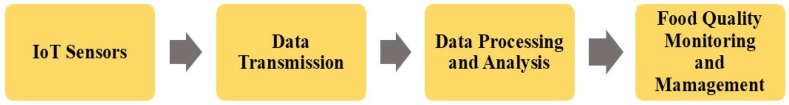
IoT framework for food quality and safety monitoring.

In particular, with the recent advancement of IoT, the field of consumer sensory science has enabled real-time product monitoring, quality control during production, and stability assessment during storage, thereby facilitating the establishment of a more precise and systematic product manufacturing framework (Figure 6). The previous studies presented in Table 11 demonstrate that IoT technologies play an important role in areas such as food freshness evaluation, quality control, and storage environment monitoring within the food industry.

### 3.5. Comparison of Digital Technologies in Food Industry

Advancements in digital technologies—such as AI, XR, biometrics, and digital sensors—are introducing new methodological approaches to sensory science. Across sensory evaluation and the broader food industry, each technology is applied in distinct ways, and when integrated, they provide complementary strengths that enhance the precision, depth, and overall effectiveness of sensory analysis.

In sensory science, AI technologies primarily include ML, text-mining–based NLP, and LLMs. Despite limitations such as the risk of overfitting and reduced predictive performance resulting from single-method data collection [40,41,42,43], AI has been applied not only to analyze and predict consumer data but also for various purposes such as improving product quality through taste and quality prediction and identifying peptides that contribute to flavor perception [10]. AI learns patterns from large-scale data to construct models capable of prediction and interpretation and can be defined as a technology that mimics human cognitive processes [9,10].

Extended Reality technologies play an important role in capturing consumers’ sensory responses as they occur in natural environments by narrowing the gap between controlled laboratory settings and real-world consumption contexts [46,52,65]. In sensory evaluation research, XR has been applied for various purposes, including immersive sensory and consumer testing, evaluating contextual effects on perception, and constructing virtual food-choice environments [46]. Although XR still faces limitations such as discomfort caused by HMDs, cognitive overload, and high implementation costs, its accessibility and technological sophistication continue to improve [65]. XR can therefore be described as a technology that simulates real consumption environments and provides immersive experiences through virtual spaces [52,65].

In sensory evaluation research, biometric technologies measure a wide range of consumer responses, including emotional reactions, physiological and neurological responses, attention levels, and perceptual states, thereby providing additional consumer information [52,81,86,155]. Although limitations such as restrictions from wired devices, limited movement, and relatively small participant groups still exist [93,155,156], biometric technologies are expected to become a key tool in future sensory evaluation research due to their ability to capture consumer responses in a more comprehensive manner [22]. Thus, biometric technologies can be defined as major emerging digital tools that enhance the understanding of consumer behavior and preferences based on physiological responses and strengthen the prediction of consumer reactions [52,53].

Digital sensor technologies—including robotics, E-nose, E-tongue, and IoT systems—are increasingly being utilized in sensory evaluation research, with their applications expanding across the broader food industry. Robotics, based on human–robot interaction, is widely applied not only in sensory evaluation but also throughout various stages of food production processes [164]. E-nose and E-tongue technologies, which mimic human sensory organs, enable the objective measurement of the physicochemical properties of food products [52]. IoT technologies are primarily used for real-time monitoring and quality management within the food supply chain, and they play an important role in enhancing product quality through automated systems that provide rapid feedback [168,171].

Despite their expanding use, digital sensor technologies still have limitations that prevent them from fully replacing human sensory evaluation in both sensory research and the food industry. To overcome these limitations, it is necessary to utilize integrated data derived from various digital sensor technologies [52,164]. In conclusion, digital sensor technologies can be primarily defined as tools that complement human sensory evaluation and enhance product quality in sensory assessment and across the broader food industry [52].

Table 12 summarizes the advantages, limitations, and application areas of state-of-the-art digital technologies in the food industry.

## 4. Integrated Application of Advanced Sensory Evaluation Technologies

### 4.1. Digital Sensing Technologies and Machine Learning

Electronic sensory systems offer the advantage of being free from human subjectivity while detecting subtle sensory characteristics that are imperceptible to humans and generating chemical data that contribute to sensory attributes [175]. Accordingly, in the field of consumer sensory science, such systems have been utilized as complementary tools to human-based sensory evaluations, and recent research has increasingly focused on combining these systems with ML technologies for quality prediction, classification, and as potential alternatives to conventional sensory evaluation data. Table 13 presents a summary of previous studies that utilized electronic sensory sensors as input data for ML models.

### 4.2. Integrating Advanced Sensory Evaluation Technologies

Although both XR and AI are based on computer technologies, they differ in their applications: XR provides virtual environments that offer consumers experiences similar to real-world settings, while AI replicates the structure of the human brain through computational models to collect and process vast amounts of data [179]. Recently, research has increasingly focused on integrating IoT technologies—which enable real-time monitoring and data automation—with XR or AI systems, along with the convergence of various other sensory evaluation technologies.

In particular, studies that collect sensory data through robotics and electronic sensory systems and apply them across different sensory evaluation frameworks are now reaching a stage where the automation of sensory assessment can be practically realized. Therefore, the integrated approach of these technologies can enhance the precision of traditional sensory evaluation and more realistically simulate and analyze consumers’ product experiences, indicating high potential for practical application in the future food industry. Table 14 presents a summary of previous studies that integrated multiple advanced sensory evaluation technologies.

## 5. Developments in Sensory Software

Traditionally employed paper-based questionnaires in sensory evaluation require considerable time and labor to review responses and to enter and organize data after the evaluation has concluded [184]. These procedures, depending on the tools and modes adopted, increase the researcher’s burden and reduce the efficiency of data management. To overcome these limitations, the use of sensory-evaluation software has been proposed, and it has recently been reported that, when combined with electronic survey tools, the efficiency of data collection and analysis can be improved [184].

According to the classification framework presented in prior research [185], currently commercialized sensory-evaluation software can be grouped into three functional categories. The first category comprises integrated programs that support the entire workflow—from experimental design and panel management to data analysis and reporting—including Compusense (Compusense 25.0.30), Fizz, RedJade, EyeQuestion (version6), and SIMS Sensory Software Cloud (version 6); more recently, cloud-based extensions such as Compusense Cloud have also been utilized. The second category includes programs specialized for statistical analysis and visualization, among which Senstools, XLSTAT, and SensoMineR are widely used; multivariate analysis tools such as Unscrambler X and FactoMineR likewise support the interpretation and visualization of complex sensory data. The third category consists of software aimed at evaluating panel performance and test reliability—such as PanelCheck, V-Power, and SensCheck PanelView—which analyze assessor consistency, discriminative ability, and repeatability to enhance panel operations and improve test reliability.

Recently, sensory-evaluation software and platforms incorporating advanced technologies have been developed. Among these, the BioSensory App simultaneously presents questionnaire items on a tablet PC and records participants’ responses on video, which are analyzed by computer-vision algorithms and ML techniques to derive biosignals such as affective and physiological responses [186]. Ref. [186] proposed a system that integrates eye tracking and affective biosignals using the BioSensory App, and [187] employed it to collect and analyze self-reported responses and biosignals in immersive terrestrial and space environments, thereby demonstrating new possibilities for sensory evaluation. In parallel, iMotions is a platform capable of integrative acquisition and analysis of multiple biosignals—EEG, GSR, HRV, and eye tracking—and has been widely utilized to elucidate relationships between sensory stimuli and physiological responses.

Andrade and BastosIn addition, the Affectiva Affdex SDK automatically analyzes FE to quantify consumers’ emotional responses; ref. [146] used Affectiva Affdex SDK 4.0 together with iMotions to capture both implicit and explicit affective responses during children’s food tasting. Alpha MOS’s dedicated software for electronic-nose and E-tongue systems (AlphaSoft) provides an integrated platform for instrument control, data acquisition, preprocessing, and statistical analysis, and is employed to quantify flavor attributes and explore quality differences in foods. Recent studies have attempted to combine data collected with AlphaSoft with algorithms or to link such data to IoT-based production monitoring systems. Ref. [188] used AlphaSoft to fuse GC–E-nose, E-tongue, and electronic eye (E-eye) measurements for a comprehensive evaluation of the aroma, taste, and color of black tea.

Furthermore, Ref. [177] applied NIR and E-nose data for coffee samples, combined these with HS-SPME–GC–MS and QDA, and implemented regression-based ML (ANN) models to predict volatile compounds and sensory attributes according to fermentation and roasting levels. XR-based sensory-evaluation software remains at an early research stage and relies more on research-oriented platforms designed by investigators than on commercial products. Ref. [55] constructed a virtual sensory-evaluation booth to examine differences from traditional evaluation environments, and [64] implemented an olfactory identification procedure using a Unity3D-based VR platform, thereby indicating new possibilities for the assessment of olfactory stimuli.

Therefore, these contemporary software systems enable multidimensional data collection and analysis through the convergence of AI, ML, biosensing, digital sensors, and XR technologies, extending the scope of sensory evaluation from conventional questionnaire-based approaches to immersive and automation-oriented paradigms. Moreover, as sensory-evaluation software has been combined with electronic survey tools and remote-session capabilities, it has evolved to support online assessments that minimize temporal and spatial constraints. In practice, ref. [189] compared three remote consumer-evaluation methods and examined differences in acceptability, engagement, and practicality, while [190] assessed the validity and reproducibility of remote testing relative to laboratory testing and proposed guidelines for remote operation. These studies indicate that electronic-survey and remote-session functionalities constitute important development trajectories for sensory software.

## 6. Ethical Considerations

### 6.1. AI Technology

Artificial intelligence has emerged as a new paradigm that can advance traditional sensory evaluation methodologies. Human perception and description of food “taste” lack absolute reference points and often exhibit diverse and unpredictable patterns. Given this inherent complexity, the adoption of AI for large-scale data analysis has become an increasingly effective methodological approach for sensory evaluation. However, the implementation of AI in the field of consumer sensory science requires careful ethical consideration.

Ref. [29] pointed out that when sensory data are applied to ML models, data collected within specific countries or cultural contexts cannot be universally generalized across all markets. In addition, according to [191], such as ChatGPT may be influenced by human-generated training data, potentially resulting in biased or distorted outcomes. The authors further raised concerns about data security and the risk of personal privacy violations during model training [191,192].

As the era of AI progresses, the protection of personal privacy has become an increasingly critical issue, suggesting that the indiscriminate adoption of AI technologies may elicit consumer resistance due to ethical concerns [192]. Moreover, when collecting sensory data for AI-based applications, it is essential to account for cross-national and cultural differences. Since AI cannot fully capture the diversity and complexity of human expression, continuous monitoring and methodological refinement are necessary to ensure the reliability and validity of research outcomes.

### 6.2. XR (VR, AR, MR)

While XR offers new opportunities for sensory evaluation, it simultaneously entails a range of ethical concerns, including privacy protection, physical and psychological harm, bias, abuse within virtual spaces, and accessibility.

In particular, in sensory evaluation, VR continuously collects not only gaze, motion, and usage patterns but also physiological signals such as HR and SC and analyzes these data to tailor virtual environments to user behaviors [193]. If nonverbal cues that readily identify individuals are collected over extended periods and the transparency of their processing and sharing is not ensured, the risk of constructing profiles of individuals’ preferences and behaviors increases [193]. Moreover, advanced features such as location-tracking cameras and built-in microphones have been identified as additional concerns, insofar as some data may be captured even when these functions are nominally deactivated [194]. Accordingly, a privacy framework that centers on purpose limitation, data minimization, secure storage, controls on third-party transfers, transparent notice, and explicit consent is required. Furthermore, the participant information sheet and informed consent form should include clear language specifying the types of information that will be collected and recorded in the XR environment, the planned data-retention period, the procedures for data processing, and whether and under what conditions secondary use of the data will be permitted. In addition, the consent procedure should explicitly inform participants of their right to withdraw from the study at any time, whether and to what extent they may request deletion of their personal data, and the limitations of any de-identification measures applied. Physical and psychological harm likewise constitute important ethical considerations.

Physically, immersive systems pose risks such as disorientation and collisions; psychologically, users may experience cybersickness or excessive strain from intense affective stimuli. To minimize these risks, standardized procedures—pre-screening, safe-environment setup, conservative exposure scheduling (short sessions with breaks), real-time monitoring, predefined stopping criteria, and post-exposure recovery support—are essential [50]. Finally, high equipment costs, language proficiency, physical conditions, and cognitive demands can restrict participation, undermining sample representativeness and exacerbating economic and digital divides [193,195]. Consequently, accessibility-first design and the reduction in participation barriers are needed to ensure the inclusion of diverse users [196]. Therefore, the responsible implementation of XR-based sensory evaluation requires the institutionalization of minimum standards for privacy, safety, and accessibility, as well as the explicit documentation of policies on data-retention periods, de-identification, and secondary use of data. In addition, governance structures should be in place to systematically assess and disclose sample representativeness and potential sources of bias.

### 6.3. Biometrics Technology

Biometric technologies and traditional explicit sensory evaluations help prevent cognitive bias among study participants [195] and hold potential as alternatives to conventional sensory evaluation [197]. However, they also present ethical limitations such as the risk of participant re-identification, lack of inclusivity, and insufficient standards for risk assessment.

First, there is a possibility of re-identifying research participants. In particular, it has been pointed out that individuals can be identified through gaze response patterns such as eye-tracking and pupil size [198], raising concerns about potential invasion of participant privacy. As biometric recordings inherently capture unique physiological and behavioral patterns, consent forms should clearly specify the types of data collected, the purposes of analysis, conditions for secondary use, and participants’ rights to request data deletion [198,199,200]. Second, biometric technologies may introduce sample bias due to issues of inclusivity regarding people with disabilities, specific ethnicities, or demographic groups. One study reported that certain participants with disabilities experienced considerable discomfort when removing their glasses, and that glare reflections while wearing glasses interfered with the iris recognition process [201].

Additionally, ref. [199] found differences in accuracy related to participants’ race during facial recognition data analysis. Similarly, ref. [202] revealed that at low false match rate (FMR) levels, age-related loss of skin elasticity in older adults can degrade image capture performance. Third, existing Institutional Review Board (IRB) or human-subject research protocols are insufficient for assessing risks associated with biometric data. The current IRB framework was not designed for large-scale data collection and may overlook issues such as long-term data storage. Accordingly, data retention should be restricted to the minimum period necessary to fulfill the research objectives [198]. It has been suggested that including experts in biometric technologies and cybersecurity on IRB review panels could enhance the effectiveness of risk assessment for biometric research [200].

### 6.4. Digital Sensor

Advancements in robotics and electronic sensory technologies have established these systems as complementary tools to human-based sensory evaluation; however, several factors must still be considered before they can fully replace human sensory assessment. Ref. [164] reported that participants who tasted coffee extracted by a robotic barista exhibited a significant reduction in their food technology neophobia scale scores compared with those who tasted coffee brewed by a human barista. Emotional response analyses further indicated that consumers experienced distinct emotional states depending on whether the coffee was prepared by a robot or a human. Similarly, ref. [203] demonstrated that electronic sensory devices such as E-tongues were able to detect wine flavors with greater sensitivity than human sensory panels.

Nevertheless, the authors pointed out that certain flavor attributes perceptible to humans may not be captured by the E-tongue. These findings suggest that when commercializing robotic and electronic sensory technologies, it is essential to address potential issues such as consumer unfamiliarity and resistance. Moreover, human sensory evaluation remains indispensable for comprehensively capturing the subtle and multidimensional aspects of sensory perception. Therefore, when applying electronic sensory systems to ML models, it is essential to incorporate cross-validation procedures to verify the degree of agreement between sensor-based outputs and human sensory data [18].

In the food industry, the IoT demonstrates outstanding capabilities in large-scale data collection, real-time monitoring, and automation; yet several challenges persist. IoT systems cannot function independently, as they require the acquisition of vast amounts of data, which introduces multiple concerns. According to [171], major issues include the protection of personal data collected during consumer monitoring processes, increased consumer burden caused by higher supply chain management costs, and the need to comply with legal and regulatory requirements related to food certification and safety management.

To effectively and responsibly implement IoT technologies in the food sector, strict management of personal data is essential to build consumer trust, while strategies are needed to mitigate the financial burden placed on customers. Furthermore, as emphasized by [204], the establishment of formal governmental regulations and policy frameworks is crucial for addressing potential ethical issues and ensuring that systematic procedures are in place to manage the broader challenges associated with IoT adoption in food production and quality control.

## 7. Future Directions

Future sensory evaluation research is expected to evolve into a more sophisticated and contextually realistic assessment environment through the convergence of emerging technologies such as AI and ML, XR, biometrics, and IoT sensor networks. Conventional sensory analysis and consumer evaluation have inherent limitations in ecological and external validity due to the constraints of controlled laboratory settings [205]. In contrast, XR-based environments enable researchers to control identical environmental variables for participants while providing higher ecological validity than conventional laboratory settings, thus offering the potential to overcome the limitations of traditional sensory evaluations [46].

Biometric technologies quantify participants’ unconscious emotional responses to food by measuring physiological signals, thereby compensating for cognitive biases that arise when relying solely on self-reports [156]. They also provide deeper insights into the psychophysiological processes associated with food preference and intake [206]. Ref. [81] suggested that the value of biometrics in sensory and consumer research will increase when applied under more realistic research designs.

AI-based sensory and consumer science research utilizes ML to integratively analyze sensory data such as food acceptability, sensory attributes, and electronic sensor signals, enabling predictive modeling and automation of the evaluation process. Furthermore, AI plays an important role as an effective tool for collecting, exploring, and analyzing both instrumental and human data in sensory and consumer research [207]. Technological advancements in AI and ML are expected to move beyond fragmented analyses of individual evaluation parameters toward the integrated interpretation of consumers’ multidimensional data on food.

The incorporation of IoT sensor networks enables the interconnection of various sensor nodes that collect data on environmental variables, biosignals, and product characteristics through wireless communication systems, thereby facilitating real-time data collection, transmission, and analysis [208]. The application of IoT sensor networks is expected to create evaluation environments capable of real-time control of physical factors such as temperature, noise, aroma, and illumination, while linking these data with biometric signals to quantitatively correct the effects of environmental variables.

Research in sensory and consumer science utilizing emerging technologies should move beyond the independent use of each technology and progress toward building an intelligent and adaptive consumer evaluation system integrated within a unified framework. Such a system would employ AI to collect and analyze data, use XR to enhance ecological validity, utilize biometric data to increase the predictability and reliability of results through unconscious emotional responses, and integrate diverse data via IoT sensor networks to enable more precise environmental control during evaluations.

Moreover, future research should examine the integration of advanced computational approaches—such as foundation models, multimodal generative models, and reinforcement learning frameworks—into sensory and consumer evaluation pipelines. These methods may enable autonomous scenario adaptation, personalized stimulus optimization, and real-time adjustment of XR environments based on biometric feedback, ultimately supporting the development of closed-loop, intelligent-adaptive evaluation systems [209,210].

## 8. Conclusions

This review systematically summarized and organized the latest research in sensory and consumer science incorporating digital technologies into four categories: AI/ML, XR, biosensing, and digital sensors. Furthermore, it examined the literature on data integration and analysis through AI and ML, quantification of unconscious responses and reduction in self-report bias through biosignals, enhancement of ecological validity and immersion through XR, and precise control and real-time data acquisition through digital and IoT sensors. Based on these findings, an integrated framework linking multimodal signals with consumer perception was proposed. Additionally, it was inferred that the convergence of these technologies will promote the efficiency, quantification, and real-time capability of future sensory evaluations while improving ecological validity. Future research should extend applications to a wider range of food products and systematically investigate the methodological, ethical, and institutional validity and reliability surrounding these convergent technologies, with a view toward the development of emotion-based and personalized foods.

## Figures and Tables

**Table 1:** Summaryof previous studies employing ML across various food categories and the ML algorithms used.

Technology	Food	Objective	Key Findings/Summary	AI/ML Algorithms (Validation Protocol)	Reference
**AI/** **ML**	Red wine	To predict wine sensory attribute from simple chemical data (Voltammetry, EEM, absorbance) using ML and PLS.	ML methods (RF and XGBoost) accurately predict wine mouthfeel from simple chemical data and outperform PLS (RF: R^2^ = 83~85%/RMSE 0.280~0.354, XGBoost: R^2^ = 91~92%/RMSE 0.206~0.230), offering a fast and low-cost approach for sensory prediction.	[RF, XGBoost] **-** DS: *n* = 30 **-** OR: RF = low/XGBoost = high (K-fold cross-validation)	[14]
	Jiang -Flavor Baijiu (JFB)	To develop a predictive strategy for the global aroma profile of JFB, the present study integrates volatile compound data with ML algorithms.	ML(NN) showed the best performance in predicting JFB aroma (R^2^ > 0.99), identifying 18 key flavor compounds, which were further validated through spiking, omission tests.	[NN, DT, PLS, RF, SVM] **-** DS: *n* = 27 (dataset: *n* = 96) **-** OR: RF = low/NN, DT, PLS, SVM = high (5-fold cross- validation method)	[17]
	Meat	To identify pork patty samples containing different levels of chicken adulteration. using ML techniques was the aim this study.	BP-ANN demonstrated the highest accuracy in predicting chicken adulteration levels in pork patties (99.52%), and SHAP analysis identified key discriminant indicators (e.g., Thr, C *, His).	[PLS-DA, SVM, BP-ANN] **-** DS: *n* = 300 (Dataset: *n* = 43) **-** OR: PLS-DA = low to medium/SVM = medium to high/BP-ANN = high (SVM: 5-fold cross-validation method)	[20]
	Drinking Water	To develop a reliable predictive model for drinking water flavor by integrating diverse water quality indicators with ML techniques.	XGBoost showed the highest accuracy in predicting drinking water flavor (R^2^ = 0.916, RMSE = 0.482), and SHAP analysis identified key water-quality indicators. A simplified model using only 10 parameters also maintained strong performance.	[PLS, ENR, SVR, RF, DT, XGBoost] **-** DS: *n* = 78 (dataset: *n* = 110) **-** OR: PLS, ENR, RF = low /SVR = medium to high /DT, XGBoost = high (5-fold cross- validation method)	[21]
	Freeze- Structured Meat	To optimize the PPI-ISP-VWG blend using RSM and evaluate freeze-structured plant-based meat with chicken-like texture.	ML models accurately predicted meat analog properties, with top performance from Gradient Boosting (hardness: R^2^ = 0.986, RMSE = 24.698), AdaBoost (springiness = R^2^ = 0.940, RMSE = 0.019), and XGBoost (water activity: R^2^ = 0.985, RMSE = 0.002).	[DT, KNN, XGBoost, RF, Gradient Boosting, AdaBoost] **-** DS: *n* = 16 **-** OR: RF = low/AdaBoost = medium to high/DT, KNN, XGBoost, Gradient Boosting = high (RMSE and R^2^ values for cross-validation)	[22]
	Beer	To develop an NIR-based and ML-driven method for beer authentication, quality evaluation, and control through the bottle.	NIR spectroscopy combined with ANN models accurately authenticated beer, predicted sensory attributes and volatile compounds through unopened bottles, offering a fast, non-destructive tool for quality control and fraud detection. (model 1: 99%, model 2: R = 0.92, model 3: R = 0.94)	[ANN] **-** DS: *n* = 25 **-** OR: ANN = high (Neuron trimming test)	[23]
	Fermented pomegranate juice (FPJ)	To use ML and SHAP analysis to identify key physicochemical factors influencing sensory preference in FPJs.	Gradient Boosting achieved the highest accuracy in predicting FPJ preference, and SHAP analysis identified TSS, CD, and LAB as the key influencing features. (CPS/WPS model: R^2^ = 0.81)	[LR, RR, KNN, SVR, RF, AdaBoost, Gradient-boosted aggregation, ANN] **-** DS: *n* = 90 **-** OR: LR, RR, RF = low/ KNN, SVR, AdaBoost = medium to high/ Gradient-boosted aggregation, ANN = high (3-, 5- and 10-fold cross-validation)	[12]

EEM = Excitation-Emission Matrix; RMSE = Root Mean Square Error; DS = Data Size; OR = Overfitting Risk; NN = Neural Network; BP-ANN = Back-Propagation Artificial Neural Networks; SHAP = Shapley Additive Explanations; PLS-DA = Partial least squares discriminant analysis; ENR = Elastic Net Regression; PPI = Pea Protein Isolate; ISP = Isolated Soy Protein; VWG = Vital Wheat Gluten; RSM = Response Surface Methodology; NIR = Near-Infrared; TSS = Total Soluble Solids; CD = Color Density; LAB = Lactic Acid Bacteria; LR = Linear Regression; RR = Ridge Regression.

**Table 2:** Summary of previous studies on NLP-based LLMs in sensory/consumer research and the food industry.

Technology	Food	Objective	Key Findings/Summary	AI/ML Algorithms	Reference
**NLP/** **LLM**	Madeleine	To evaluate how different FC data formats (words vs. sentences) and preprocessing methods affect the quality and reliability of results.	ChatGPT and the expert system performed well on word-based FC data but showed lower performance than human experts on sentence-based FC data, and preprocessing methods led to large differences in reproducibility and discriminative power.	[NLP, LLM]	[30]
	Wine	To demonstrate how NLP and ML techniques can be used to analyze expert-written Bulgarian wine descriptions and extract patterns related to wine quality and style.	NLP and ML enabled automatic extraction of quality and style patterns from Bulgarian wine descriptions, with BERT-based models showing high performance in predicting wine style and ratings (R^2^ = 0.643~0.656).	[BERT, SVM, RF, XGBoost, MLP] **-** Dataset: *n* = 5807	[29]
	Chocolate brownies	To evaluate the potential use of Chat GPT as a sensory evaluator for hypothetical chocolate brownie formulations.	ChatGPT provided highly positive and overly favorable sensory descriptions for all brownie formulations, showing sentiment bias and requiring validation against human sensory panels.	[NLP, LLM]	[31]
	Sustainable protein foods	To investigate how LLMs can support sustainable food development by evaluating their performance across key design and prediction tasks and integrating them with optimization methods.	LLMs, when combined with optimization techniques, can generate food choices that reduce greenhouse gas emissions by up to 79% while maintaining user satisfaction, demonstrating their potential to support sustainable food design.	[LLM]	[32]
	Sweetness	To analyze sweetness levels, liking, and ingredient information from online food reviews to gain insights into sensory nutrition and identify opportunities to reconcile the palatability-healthiness tension.	Oversweetness found in 7–16% of sweetness-related reviews and was consistently linked to lower liking, indicating a clear opportunity for developing reduced-sweetness product versions. (XGBoost accuracy: 79–84%)	[NLP, XGBoost] **-** Dataset: *n* (total) = about 550,000 (Sweetness - related reviews)	[26]
	Whisky	To identify and extract unique sensory descriptors from existing whisky reviews to build a flavor language.	LSTM and GloVe-based DL models accurately extracted whisky flavor descriptors from review texts with 99% accuracy, demonstrating that a flavor language can be programmatically learned.	[NLP, LSTM, GloVe] **-** Dataset: *n* = 8036 (English whisky reviews)	[24]

FC = Free Comment; BERT = Bidirectional Encoder Representations from Transformers; MLP = Multi-Layer Perceptron Regressor; LSTM = Long Short-Term Memory; GloVe = Global Vectors for World Representation.

**Table 3:** Summary of previous studies on AI/ML applications in molecular docking and physicochemical-based food research.

Technology	Food	Objective	Key Findings/Summary	AI/ML Algorithms (Validation Protocol)	Reference
**ML**	Oyster	To rapidly identify oyster-derived umami peptides using ML and to clarify their umami and salt-enhancing mechanisms through molecular docking and sensory analysis.	Three oyster-derived umami peptides were identified using ML, and molecular docking confirmed their binding to T1R1/T1R3 and TMC4, revealing strong umami and salt-enhancing properties.	[iUmami-SCM, Umami_YYDS, TastePeptides -DM] **-** Dataset: *n* = 159	[36]
	Saltiness	To predict the saltness-enhancing intensity of savory odorants using an XGBoost regression model and to elucidate their structural and receptor-binding mechanisms through SHAP analysis and molecular simulations.	XGBoost accurately predicted saltiness-enhancing intensity (R^2^ = 0.96), SHAP identified key structural groups (phenyl, aldehyde), and molecular simulations revealed key OR1A1/OR1D2 binding sites explaining odor-induced salt enhancement.	[XGBoost] **-** Dataset: *n* = 81 **-** OR: high (5-fold cross-validation)	[16]
	Sufu	To elucidate the formation mechanism of umami peptides during sufu fermentation and to establish a rapid screening model using peptidomics, ML, and molecular docking.	Peptidomics and ML identified 637 umami peptides, and molecular docking with sensory validation confirmed five novel peptides that bind T1R1/T1R3 and impart actual umami taste.	[Umami-MRNN, UMPred-FRL, Umami_YYDS] **-** Dataset: *n* = 637	[35]
	Sausage	To develop an integrated DL-based framework combined with metagenomics and molecular docking to efficiently predict, screen, and validate potential umami peptides in fermented sausages.	Integrated DL and metagenomics enabled high-throughput screening of umami peptides, identifying top candidates that showed stable T1R1/T1R3 binding and strong umami taste validated by molecular docking, MD simulation, and sensory evaluation. (Accuracy: CNN = 82.4%, Transformer = 79.4%, LSTM = 81.4%, Attention = 81.6%)	[CNN, Transformer, LSTM, Attention architectures] **-** Dataset: *n* = 508 **-** OR: high (80/20 split with a balanced da taset and an all-model consensus ensemble)	[37]
	Pixian Doubanjiang (PXDB)	To identify umami peptides in aged PXDB using ML and molecular docking, and to elucidate their sensory mechanisms and biosynthetic pathways.	ML identified 69 potential umami peptides from PXDB, with VEGGLR confirmed to have a very low umami threshold and strong T1R1/T1R3 binding, while PTM profiling suggested regulatory roles in umami peptide biosynthesis.	[Umami-MRNN] **-** Dataset: *n* = 117	[38]

SHAP = Shapley Additive Explanations; OR = Overfitting Risk; CNN = Convolutional Network; LSTM = Long Short-Term Memory; PTM = Post-Translational Modification.

**Table 4:** Descriptions of VR, AR and MR (Adapted from [57], with permission from MDPI, ©2020).

	Virtual Reality	Augmented Reality	Mixed Reality
Display device	Special HMD or smart glasses required.	Smartphones, tablets, AR glasses or headsets (optional).	HMD or AR glasses (optional handheld or projection devices).
Image source	Computer graphics or real images produced by a computer.	Combination of computer-generated images and real-life objects.	Combination of computer-generated images and real-life objects.
Environment	Fully digital.	Physical surroundings with overlaid virtual content.	Real and virtual elements coexist and interact in real time
Perspective	Virtual objects adjust in size and position according to the user’s viewpoint in the virtual world.	Virtual objects align with the user’s real-world viewpoint.	Virtual objects align and interact with the user’s real-world viewpoint.
Presence	Feeling of being transported somewhere else with no sense of the real world.	Feeling of still being in the real world, but with new elements and objects superimposed.	Feeling of still being in the real world, but with new elements and objects superimposed.
Awareness	Highly rendered virtual objects may be indistinguishable from reality.	Highly rendered virtual objects may be indistinguishable from reality.	Virtual objects may be indistinguishable from real ones and can be manipulated as part of the physical environment.

**Table 5:** Summary of literature on VR, AR, and MR.

Technology	Food	Objective	Key Findings/Summary	Reference
**VR**	Virtual cake	To assess the effects of VR on visual liking and hedonic responses to cakes across two immersive, photogrammetry-based contexts.	No main effect of context on liking; visual liking differed significantly by the context–cake interaction, age, and subjective hunger.	[62]
	Chocolate biscuits, orange juice	To design a VR-based sensory booth to complement sensory evaluation and expand applications in sensory science.	Feasible VR-based sensory booth enabling multiple sensory methods for evaluation and perception research.	[63]
	Scent sticks	To test whether VR food imagery modulates odor identification and perception via a VR-integrated olfactory task.	Olfactory augmentation in VR heightened presence, enhanced recall, improved comfort and affect, and influenced consumer behavior.	[64]
	Bakery items, Scented sticks	To evaluate a VR-based sensory laboratory integrating conventional sensory methods to examine differences in consumer responses.	SSQ, virtual reality sickness questionnaire and virtual reality neuroscience questionnaire scores indicate the virtual sensory laboratory is suitable for consumer sensory evaluation.	[65]
	Granola bar	To assess how consumption-environment personal relevance (usage frequency) shapes perception and acceptance.	Personal relevance increased data repeatability, yielding more reliable consumer insights.	[66]
	Apple juice	To compare presence, liking, beverage desire, intake, and choice across real, lab, and two immersive contexts.	360VR induced stronger café presence than a picture-based context, while liking remained comparable to laboratory ratings.	[7]
	Sandwich	To compare responses across different experimental setups.	Immersion ranked: real-life > simulated environments > scenario-based booth; pattern consistent with external validity.	[48]
**AR**	Chicken meal	To assess effects of AR simulated control and environmental embedding on mental imagery, evaluation ease, liking, and purchase intention.	Environmental embedding’s effect on product liking was fully mediated by mental imagery quality (no direct effect).	[67]
	Dessert	To assess whether AR superimposition enhances mental simulation, increasing desire and purchase intention.	AR raised mental simulation, which mediated higher desire and purchase likelihood.	[60]
	10 different food images	To develop and validate an AR tool for food portion estimation.	AR improved portion-size estimation accuracy.	[68]
	Yogurt	To evaluate how AR environments influence consumer sensory responses to different yogurts.	Significant yogurt–environment interaction for appearance, flavor, sweetness, mouthfeel, aftertaste, and overall liking.	[69]
	Beverage	To build and evaluate a wearable AR–olfaction system to test how visual and scent cues modulate taste perception.	Olfaction exerted a stronger influence on flavor perception than vision.	[70]
**MR**	Snack and real foods	To assess utility and ecological validity of an HMD-passthrough MR app for interacting with real foods.	Experts rated the virtual restaurant more acceptable than a sensory booth, but less acceptable than a real restaurant.	[71]
	Tea break snack	To examine how consumption context—including MR—shapes consumers’ emotional responses to tea-break snacks.	Incorporating context is crucial for consumer emotional-response data collection.	[72]
	Tea break snack	To compare consumer affective responses to snacks across a sensory booth, an MR-evoked café, and a real café to assess MR’s ecological validity.	Affective ratings in the MR café matched the real café (*p* ≥ 0.10), supporting MR as an ecologically valid setting for consumer testing.	[47]
	Snack foods, Beverages	To develop an MR HMD–camera computer vision system to detect diet-related actions and trigger real-time visual interventions that promote healthier choices.	Current neural networks achieve high-accuracy food item detection in real-world settings.	[73]

**Table 6:** Summary of literature on nerve and brain activity.

Technology	Food	Objective	Key Findings/Summary	Reference
**EEG**	D-limonene, essential oils	To compare the brain’s sensory and cognitive responses to various citrus flavors using EEG.	Left-right asymmetry of alpha waves and intensity of delta waves in the prefrontal cortex showed a significant correlation with liking ratings for citrus flavors.	[92]
	Baijiu	To evaluate the predictive validity of brainwave attentiveness and facial expressions for pairing preferences.	Sweetness and saltiness were key drivers of preference; white liquors with elegant flavors matched sweetness, while spicy ones paired well with diverse tastes (umami, saltiness, sweetness).	[95]
	Protein chocolate milk	To compare the effects of health- and taste-related perceptions on explicit and implicit food preferences.	EEG and fMRI results indicated that health-related perceptions reduced explicit preferences compared to taste-related perceptions, but did not affect implicit preferences.	[96]
	Marinated beef	To examine neural responses to different cooking methods using EEG.	High-heat cooking elicited stronger α, β, and γ activity, linked to pleasure, appetite, and cognitive engagement.	[97]
	Food samples with unpleasant/pleasant aromas	To integrate brainwave technology and pattern recognition techniques to provide objective, quantified physiological data reflecting responses to odor stimuli.	Developed an experimental paradigm capable of collecting olfactory EEG responses to eight distinct odors and proposed a new olfactory perception dimensional space theory.	[66]
	Coffee	To examine gender differences in coffee preference based on GI information using EEG analysis.	Men preferred coffee with GI information, while women favored coffee without it; EEG results contrasted with self-reported preferences.	[98]
	Coffee	To predict the sensory characteristics of coffee using EEG and ML technologies.	Signals from the parietal lobe, central lobe, and frontal lobe regions showed the highest predictive power.	[99]
	Alcohol solutions and Baijiu	To compare brain responses to white liquor and alcohol of equal concentration.	White liquor showed significantly higher brain signal activity (increased δ, α waves and heightened frontal lobe, parietal lobe, right temporal lobe).	[100]
**fNIRS**	Sucrose solutions	To identify brain regions associated with sweetness intensity and emotional value.	As sweetness increased, the number of activated channels rose from 7 to 11; a positive correlation between participants’ self-reported sweetness intensity data and implicit data.	[94]
	Chocolate	To confirm differences in brain activity between chocolate lovers and non-lovers.	The introduction of fNIRS to sensory evaluation demonstrated that sweetness and bitterness, respectively, decrease and increase neural activity.	[93]
	Thermal water, orange essential oil, mineral water	To investigate gender differences in brain responses to pleasant and unpleasant odors.	Compared with pleasant odors, unpleasant ones elicited a significantly greater increase in oxygenated hemoglobin; women showed higher fNIRS responses than men, with stronger activation to unpleasant odors.	[101]
	Distillate water and coffee	To examine the relationship between perceived bitterness and brain oxygenation changes.	Bitter samples showed ΔoxyHb increases in taste regions; women displayed higher ΔoxyHb for water, suggesting a link between vision and taste.	[102]
**fMRI**	Three solutions (sour taste, mango smell, and flavor of sour taste plus mango smell)	To identify brain regions involved in the integration of taste and olfactory signals and to clarify the neural mechanism underlying their interaction.	Sour taste and odor were integrated in the anterior insula and rolandic operculum, key regions activated during taste stimulation.	[103]
	Pleasant/unpleasant dishes	To investigate how plate design aesthetics influence consumers’ neural and emotional responses to food.	Pleasing designs enhanced product attitudes by activating reward and attention regions, while unpleasant designs triggered inhibition and rejection areas linked to negative evaluations.	[104]
	NaCl solutions	To examine how odor cues (MSG and cheddar cheese) affect preference for saltiness and related brain activation.	Saltiness preference increased with these odors; high-salt stimuli activated the rolandic operculum, while preference-related activation appeared in the rectus gyrus, medial orbitofrontal cortex, and substantia nigra.	[105]
	Marshmallow, caramel, grapefruit, quinine	To explore brain activation induced by odor stimuli related to taste perception.	Odors activated the insula and frontal operculum; sour odors showed stronger activity in the angular gyrus, orbitofrontal cortex, caudate, and nucleus accumbens.	[106]

**Table 7:** Summary of literature on autonomic nervous system responses.

Technology	Food	Objective	Key Findings/Summary	Reference
**EDA/GSR**	Sweet gums	To assess food acceptance by integrating FER, GSR, and heart rate measurements.	Integrating FER, GSR, and heart rate improved prediction of food acceptance; GSR and pulse enhanced accuracy beyond FER alone.	[118]
	Peppermint, jasmine, sweet orange, and lavender essential oils	To examine physiological responses to olfactory preferences using EDA.	EDA collected SC, respiration, and HR; olfactory preference affected respiration and HR, but not skin conductance.	[119]
	Hotdog, tofu	To investigate the relationship between food neophobia and physiological responses using SCR.	SCR positively correlated with food neophobia; elevated pre-presentation signals indicated expectancy toward food.	[120]
	Beer	To determine whether samples can be distinguished using EDA-derived skin conductance data.	Skin conductance alone distinguished samples; explicit symbolism and value showed negative correlation with EDA measures.	[121]
**ECG**	Red wine	To examine the relationship between ECG-measured emotions and sensory attributes.	ECG-measured emotions highly correlated with quantitative and hedonic sensory attributes; specific aromatic molecules induced positive or negative emotions.	[115]
	Universally/personally accepted/non-accepted solutions	To enhance understanding of consumers’ food experiences using HR, HRV, SC, and EEG measurements.	HR, HRV, SC, and EEG clarified food experience responses; non-accepted solutions increased HR and shortened SC response latency.	[116]
	Sucrose, quinine	To investigate physiological responses to expectation confirmation and violation during tasting.	HR decreased during second tasting; expectation-confirming tastes increased HR, while expectation-violating tastes decreased it; SC unaffected and lower in second session.	[122]
**Skin temperature**	Mushroom, fish, chocolate, caramel, cucumber, orange, apple	To examine how early facial and autonomic responses reflect olfactory arousal.	Early facial and ANS responses reflected olfactory arousal; explicit measures linked to conscious processing and odor salience.	[123]
	Vegetable juice	To analyze emotional and physiological indicators influencing purchase intention.	Significant differences in state anxiety inventory, negative sensory feedback, emotional quotient, and FE related to purchase behavior; negative emotions low and positive emotions high in self-reports and FE.	[124]

**Table 8:** Summary of literature on eye movements and visual responses.

Technology	Food	Objective	Key Findings/Summary	Reference
**Eye-tracking**	Orange juice	To investigate how packaging elements influence visual attention and purchase intent.	Visual attention focused on nutritional information, but purchase intent mainly driven by New Zealand logo.	[130]
	Sugar-sweetened beverages	To examine the relationship between nutritional awareness, visual attention, and purchase intention for beverages.	As awareness of nutrition has increased, visual attention to product attributes has mediated a growing preference for reduced or no sugar beverages.	[133]
	Test food on a tray	To investigate eye movement patterns and consumption behavior across age groups.	Total fixation time and frequency increased according to food preference, with the highest intake observed across all age groups.	[134]
	Beef	To assess how visual and informational attributes of beef affect consumer attention and purchase intent.	Deep red color increased purchase intent; brown color and Nellor breed decreased it; color, breed, marbling, and price affected fixation metrics.	[135]
	Apple, honey melon, chocolate, caramel	To examine how odor stimuli influence attention and food choice behavior.	More frequent selection of healthy foods regardless of odor; Longer initial attention span under healthy odor stimuli.	[136]

**Table 9:** Summary of literature on FE and responses.

Technology	Food	Objective	Key Findings/Summary	Reference
**FEA**	Added sugar and surprise flavor	To evaluate facial emotion decoding as a tool for distinguishing food samples.	Facial decoding distinguished samples through anger and disgust; it uniquely identified the effect of added sugar; and emotions were reflected in explicit evaluations.	[146]
	Energy drinks	To compare explicit and implicit emotional responses to different energy drinks.	Positive emotions were observed in both beverages; Energy Drink A elicited greater implicit emotional engagement than Energy Drink B.	[147]
	Oat bread	To analyze oral response patterns to different bread types using facial recognition.	Bread type affected chewing duration and frequency; facial recognition data aligned with explicit satiety results.	[148]
	Beef patty	To compare age-related differences in facial expressiveness during sensory evaluation.	Younger consumers showed greater facial expressiveness; blank expression most frequent, with age-related differences observed.	[149]
	Beer	To validate FE measurement for predicting beer selection.	FE metrics predicted beer choice; ‘Lip suck’ negatively and ‘Lip press’ positively influenced selection.	[150]
	Orange juice	To analyze the relationship between visual attention and purchase intention using FE and eye-tracking data.	Nutritional information captured most attention, but New Zealand logo determined purchase intent, showing attention and liking were misaligned.	[147]
**EMG**	Biscuits	To assess the feasibility of using EMG signals to evaluate chewing behavior and texture in biscuits.	EMG replicated chewing behavior; chewing time reflected texture attributes, suggesting utility for texture evaluation in baking.	[151]
	Chocolate	To analyze facial muscle activity in response to different taste profiles and chocolate preferences.	Facial muscle activity differed between bitter and sweet segments; activity varied by preferred cocoa content during consumption.	[152]
	Gel-type solid food	To investigate the relationship between muscle activity and subjective responses to solid food.	Preference, desire, and value for solid food negatively correlated with masseter muscle EMG activity.	[153]
	Pear juice	To examine the effect of organic labeling on muscle activation and consumer response.	Hyoid muscle activation observed during pre-observation of organic-labeled products; shorter reaction times for organic juices indicated label influence on preference.	[154]

**Table 10:** Summary of previous studies on robotics applications in food industry.

Technology	Food	Objective	Key Findings/Summary	Reference
**Robot**	Coffee	To compare volatile compounds (GC–MS), consumer acceptance, sensory profiles, and emotional responses between robot- and human-brewed coffee.	The study suggested that robot baristas could serve as an efficient alternative to human baristas, and indicated the potential expansion of human–robot collaborative models across the coffee and broader food industries.	[164]
	White- flesh dragon	To develop and validate a robot-based sensor system for non-destructive evaluation of texture degradation in dragon fruit.	The robot-based measurement method estimated the internal decay of dragon fruit with about 84% accuracy, demonstrating the potential for integrating robotics with sensory evaluation techniques.	[165]
	Food	To implement an automate taste system by integrating chemical sensors into a robotic finger, enabling rapid discrimination of food flavors and additives.	The robotic finger successfully distinguished various tastes from food samples, enabling rapid evaluation and suggesting the potential of robots to replace human sensory assessment.	[166]

**Table 11:** Summary of previous studies on IoT applications in food quality monitoring and preservation.

Technology	Food	Objective	Key Findings/Summary	Reference
**IoT**	Lettuce	To design and evaluate an IoT-based temperature and humidity control storage system to reduce postharvest losses and extend the shelf life of lettuce.	Application of the IoT-based smart storage system improved the shelf life and consumer preference of lettuce, suggesting its potential contribution to quality management and food loss reduction.	[169]
**IoT, ** **E-nose**	Beef	To evaluate volatile organic compound (VOC) concentrations for identifying beef spoilage levels, an IoT-based E-nose system was proposed.	The correlation between bacterial growth and VOC generation in beef spoilage evaluation was identified, demonstrating that the IoT-based E-nose system can serve as a real-time tool for food spoilage detection.	[170]
**IoT**	Bread	To control bread production quality, an integrated system combining various sensors and IoT technologies was proposed.	The integrated system combining various sensors and IoT technologies enabled faster and more efficient quality control and real-time monitoring compared to traditional food production management methods.	[167]

**Table 12:** Summary of strengths, limitations, and applications of digital technologies in food industry.

Technology	Strengths	Limitations	Applications	Reference
**AI**	Rapid analysis of large-scale data, capability to predict food taste, ability to interpret unstructured consumer expressions, analysis of consumer preference-sensory interactions.	Overfitting risk, domain shift, drift, lower accuracy with single-method data, limits of text-based review analysis, not fully representative.	Consumer preference prediction, food quality prediction, consumer data analysis, peptide screening for taste perception.	[9,10,11,24,25,26,39,40,41,42,43]
**XR**	Higher ecological validity, realistic consumption contexts, greater engagement and immersion, flexible context design.	HMD-induced fatigue/cybersickness, cognitive overload, novelty effects, high cost and low scalability.	Immersive sensory/consumer tests, eating-environment effects, virtual food-choice environments, VR-based food/nutrition training.	[39,45,47,55,57,71,72,172,173,174]
**Biometrics**	High temporal or spatial resolution, objective physiological indicators, sensitivity to subtle sensory differences, enhanced prediction of liking and choice.	Awareness of monitoring, restricted movement, intake-related artifacts, small participant cohorts, limited of wired devices, external influence susceptibility.	Measurement emotional responses, physiological-neural reactions, attention and perception, formulation-related responses, integrated experience, and measurements to predict choice behavior.	[73,77,78,80,86,87,89,90,91,102,109,117,118,120,135,149,150,151,152,153,154,155,156,157,158]
**Digital sensor**	Complements human sensory evaluation, reduces production time, improves quality control, enables system automation.	Cannot rely on data alone, cannot fully replace human sensory evaluation, high cost.	Sensory/consumer test, food quality monitoring and management.	[164,165,166,167,169,170]

**Table 13:** Summary of previous studies on digital sensing technologies and ML.

Technology	Food	Objective	Key Findings/Summary	AI/ML Algorithms (Validation Protocol)	Reference
**Digital** **sensing** **technologies/ML**	Soybean	To establish correlations between human sensory evaluations and multi-sensor instrument data using ML models, based on various commercial soybean paste products.	Using multi-sensor data and ML, the SVR model achieved the best performance (prediction set: R^2^ = 0.997, RMSE = 0.536), accurately predicting and distinguishing the sensory quality of commercial soybean pastes.	[SVR, RF, XGBoost, BRR, RR, KNN, ANN] **-** DS: *n* = 99 (Using a grid search methodology combined with 10-fold cross validation)	[18]
	Peaches	To design an integrated visuo-tactile sensing system and composite DL model capable of non-destructively predicting peach firmness while incorporating markers to improve contact-force estimation.	The visuo-tactile sensor enabled non-destructive prediction of peach firmness (R^2^ = 0.878, RMSE = 0.732) and contact force (R^2^ = 0.942, RMSE = 1.115), demonstrating strong feasibility for robotic-arm-based agricultural applications.	[SVR, KNR, CNN, CNN-LSTM] **-** DS: *n* = 1660 (Validation-set protocol)	[176]
	Coffee	To assess volatile and sensory differences between fermented and unfermented coffee using digital sensors (NIR, E-nose) and to build ML models predicting aroma compounds and sensory intensities.	Digital sensing combined with ANN models accurately predicted volatile compounds (R^(1)^ up to 0.98) and sensory descriptor intensities (R^(1)^ = 0.91), effectively distinguishing fermented from unfermented coffee across roasting levels.	[ANN] **-** DS: *n* = 16 (Validation-set protocol, Bayesian regularization, and neuron trimming)	[177]
	Baijiu	To integrate E-nose/E-tongue data with human sensory evaluations to develop ML models for predicting and classifying the flavor quality of strong-aroma Baijiu.	E-nose/E-tongue data combined with ML enabled highly accurate Baijiu flavor prediction (R^2^ > 0.999) and 100% classification accuracy, effectively distinguishing 42 strong-aroma Baijiu samples across origins, alcohol levels, and grades.	[LR, DT, RF, GBT, SVR, K NN, SVM, Naive Bayes] **-** DS: *n* = 42 (Test-set validation and model ensembling)	[175]
	Fruit juice	To fuse electronic sensory features with ANNs to model and predict human sensory attributes and hedonic responses to fruit juice.	Fused e-sensory features combined with ANN accurately predicted human sensory hedonic responses to fruit juice (best model R^2^ = 0.95, RMSE = 0.04), demonstrating strong potential to supplement human sensory evaluation.	[ANN] **-** DS: *n* = 287 (H yperparameter optimization)	[44]
	Beer	To evaluate consumer acceptance and perceived quality of beer based on visual foam characteristics and to develop an ANN model predicting liking using biometric and physical parameters.	Biometric and physical metrics integrated with visual foam attributes enabled accurate prediction of beer liking (ANN accuracy 82%), effectively distinguishing consumer preference for different beer types.	[ANN] **-** DS: *n* = 15 (70-15-15 train/validation/test split and cross-entropy validation)	[178]

(1) correlation coefficient; RMSE = Root Mean Square Error; BRR = Bayesian Ridge Regression; RR = Ridge Regression; DS = Data Size; KNR = K-Neighbors Regressor; CNN-LSTM = Convolutional Recurrent Coupling Network; NIR = Near-Infrared; LR = Logistic Regression; GBT = Gradient Boosting Tree.

**Table 14:** Summary of studies integrating advanced sensory evaluation technologies.

Technology	Food	Objective	Key Findings/Summary	Reference
**MIP, ML, ** **IoT**	Foods	To describe an IoT, MIP sensor, and ML integrated solution for improving the performance of a food spoilage detection system.	The MIP sensor detected VOCs related to food spoilage, and ML models classified freshness with up to 95% accuracy. An IoT framework enabled real-time freshness prediction and spoilage alerts.	[180]
**IoT, AI, ** **VR, ** **Biometric** **sensor**	Olfactory	To implement an immersive experience that synchronizes olfactory and visual stimuli through olfactory virtual reality system integrating VR, IoT, and AI technologies.	Cat-Seg, MAFT, and YOLOv11m-seg models performed odor classification; Cat-Seg showed the best results. An IoT device controlled chemical release, and biofeedback dynamically adjusted olfactory stimuli.	[181]
**IoT, AI**	Chicken	To develop a quality prediction model for the cold chain of chilled chicken, IoT and flexible sensors were integrated.	Environmental and physicochemical indicators (e.g., temperature, humidity, color, TVB-N) were analyzed with sensory data. Knowledge rule–based analysis enabled real-time food quality prediction.	[182]
**E-nose, ** **IoT, ** **ML**	Banana	To detect food spoilage and determine shelf life, an IoT and ML—based E-nose system was developed.	A low-cost E-nose with IoT connectivity (ESP8266, MOS-based sensors) detected fruit ripeness and spoilage. The SVC model achieved 97.05% accuracy in freshness classification.	[183]

MIP = molecularly imprinted polymer; VOCs = Volatile Organic Compounds; TVB-N = Volatile Base Nitrogen Rapid Detector; SVC = Support Vector Classifier.

## Data Availability

No new data were created or analyzed in this study. Data sharing is not applicable to this article.

## Abbreviations

AI	Artificial intelligence
ML	Machine learning
NLP	Natural language processing
LLMs	Large language models
PLS	Partial least squares
SVR	Support Vector Regression
SVM	Support Vector Machine
XGBoost	Extreme Gradient Boosting
RF	Random Forest
NN	Neural Network
KNN	K-Nearest Neighbors
ANN	Artificial Neural Network
DL	Deep Learning
RMSE	Root mean square error
DT	Decision tree
GC–MS	Gas chromatography–mass spectrometry
EEM	Excitation–Emission Matrix
ENR	Elastic Net Regression
PLS-DA	Partial least squares discriminant analysis
BP	Back-propagation
XR	Extended reality
VR	Virtual reality
AR	Augmented reality
MR	Mixed reality
HMD	Head-mounted display
EEG	Electroencephalogram
fNIRS	Functional near-infrared spectroscopy
fMRI	Functional magnetic resonance imaging
BOLD	Blood oxygenation level-dependent
HR	Heart rate
HRV	Heart rate variability
ANS	Autonomic nervous system
EDA/GSR	Electrodermal activity/Galvanic skin response
SC	Skin conductance
SCL	Skin conductance level
SR	Skin resistance
ECG	Electrocardiography
B.P.	Blood pressure
FEA	Facial expression analysis
EMG	Electromyography
IoT	Internet of Things
E-nose	Electronic noses
E-tongue	Electronic tongues
